# Hospitals of Paris

**Published:** 1853-10

**Authors:** 


THE following we find in the Paris correspondence of the *National Democrat* :—

" HOSPITALS OF PARIS.—The Director-General for Public Assistance in Paris, has just presented to the Council of Surveillance his account for 1852. The report is preceded by a remarkable *memoire*, in which M. Davenne passes in review the dispositions which have been taken in that which concerns the service *des infans trouves*, and of poor orphans. We shall return to this important part of his work, which merits an examination the more attentive, since the question *des infans trouves* is still far from being solved. We shall, however, limit ourselves to throwing a hasty glance over the report now before us, and giving to our readers, after the official figures collected and put in order by M. Davenne, the sum of Parisian misery, and the budget of public assistance.

It is not with impunity that one stops to turn over the leaves of such a book, the forty and some odd pages of statistics, the eloquence of whose figures seizes the heart and makes sad the soul.

Behold a city, the first in the world, the capital of intelligence, where a quarter part of the population go to die at the hospitals or the *hospice.* Within a year, upwards of 90,000 sick have been treated at these hospitals ; 18,500 aged and insane gathered in the *hospices ;* 17,000 to 18,000 *enfans trouves*, or orphans, maintained ; and 78,000 indigent succored ! We give an account here only of that misery which is in some sort public, recorded in the grand book of assistance, and officially recognized by the administration. Sickness, the infirmities of age, vice, and indigence, these are the four great divisions of the fatal book, which comprises a a fifth of the population of Paris.

In 1852 there has been expended 13,345,629 francs in giving assistance to all these unfortunates. In this budget, the hospitals comprise the sum of 3,801,976 francs ; in the *hospice* and houses of retreat, 3,780,249 francs ; the exterior service for the *enfans trouves* and their nurses, 1,653,584 francs ; the succor at private houses, 2,653,472 francs.

Though considerable as this budget appears, we are to remember that it is not entirely employed in the direct relief of the unfortunate. One is obliged to bring in the enormous personal expenses and of the general administration (1,587,319 francs), of buildings (502,403 francs), of diverse outlays for lodings, etc. (342,430 francs), of expenses for inspection and exploration (343,204 francs), that is to say, nearly two million eight hundred thousand francs; so that more than a fifth part is found to be absorbed in these general charges.

It is not a reproach that we would address to the administration of public assistance in Paris; we know its devotion to the unfortunate and we make it our duty to render homage to its direction and the benevolence which illumines it. We know the multiplied exigencies of the service which comprehends within its radius, since the law of the 10th January, 1849, thirty-five distinct establishments. We know as to Paris, and it is not otherwise in the departments, that the general revenues of the *hospice* of France amounts to more than fifty-four millions, and that ten millions are absorbed in administration. But though justly—for the fact which we signalize results from a general abuse and an administrative vice inherent in the institution itself—we believe it necessary to call the attention of the Minister of the Interior to one situation evidently abnormal. Administraiion costs dear in France. The country is without doubt rich enough to pay this—*bureaucratie.* But the poor! ought they not, above all, to have right to manage their own?

The population *des hospitaux* of Paris is divided among nine general hospitals: the *Hotel-Dieu*, *Saint Marguerite*, the *Pitie*, the *Charite*, *Saint Antonie*, *Necker*, *Cochin*, *Beaujon*, and *Bon Secours;* six special hospitals: *Saint Louis*, *Midi*, *Lourcine*, *Enfans Malades*, *Accouchment*, and *Clinique;* and lastly, *la Maison de Saut*, du fauborg Saint Denis. These last six establishments contained on the 1st January, 1852, 5,641 sick; and there entered during the year 34,845, making 90,486 treated during the twelve months. The number who went out for reason of being cured or other causes was 77,776; the number of deaths was 7,201; so that there remained on the last day of the year 5,509. The mean number of beds occupied during the year was 5,753.

The total number of sick-days of the inmates was 2,072,670. The mean time of sojurn in these hospitals is 24,39; the expense of treatment of each 43fr. 75c.; and the annual expense of each bed 654fr. 69c. The cost per day did not reach 1fr. 80c.

The time of the employed forms a total of 582,630 days, that is to say, two employers for seven sick.

Five *hospices: Virilesse*, men and women, *Incurables*, men and women, and *Enfans Trouves;* three houses of retreat: *Menages, la Rochefoucault, Saint Perine;* four foundations: *Boulard, Brezin, Devillas, Lambrechts:* in all twelve establishments, containing on the 1st January, 1852, 9,210 insane or aged. There entered during the year 9,290; 7,765 went out for various causes; 1,538 died; and there remained on the 31st Dec., 9,197.

The total number of days of administration to the insane, old, and infants, was 3,491,748. The days of the *employees* were 416,029; that is to say, two *employees* to seventeen subjects.

The annual expenses of each bed was 411f. 98c.; the average number of beds occupied 9,556, and the price per day 1f 13 nearly.

The mortality in the general hospitals was as 1 to 10:27; in the special hospitals as 1 to 19:44, and as 1 to 6:92 in the *Maison de Sante.* This is as 1 to 11:80 in all the establishments united.

Among the insane of the 3,662 the mortality was as 1 to 4:61; in the *hospices* and houses of retreat as 1 to 6:55, and in the founded *hospices* as 1 to 5:77; in all, as 1 to 5:93.

It is not without interest to search for the mean number of days those live admitted to these various establishments. M. Davenne has calculated that during the period of five years, from forty-four to forty-eight, the average life was five years, five months and sixteen days.

The number of *enfans trouves* and orphans received in 1852 was 3,033. Of these, 271 are supposed to be legitimate; 2,762 natural; 527 came from the *Maison d'Accouchment;* 280 from the hospitale of Paris; 2,154 were born in Paris; 244 out of it, and 98 were deposited without any cognizant mark. On the first of January there were being entertained 13,787; the number received during the year raised the sum to 17,880, and after deducting the number who left or died, there remained 13,829.

Finally, the indigent population inscribed at the *Bureaux de Bienfaisance* comprises a total of 77,999 individuals, divided in 33,741 families; 46,766 adults."

